# Changes in resilience and masculine gender role stress following 8 weeks of maximal power cycling training in male university students: a single-group pre-post study

**DOI:** 10.3389/fpsyg.2026.1876857

**Published:** 2026-07-24

**Authors:** Yang Chen, Guoyuan Huang, Yixin Zhan, Wenjun Ren, Xiaofeng Niu, Niezi Wang

**Affiliations:** 1 Xi’an Jiaotong University School of Physical Education, Xi’an, China; 2 College of Sport and Art, Shenzhen Technology University, Shenzhen, China

**Keywords:** exploratory mediation analysis, gender role stress, high-intensity interval training, male university students, resilience

## Abstract

Male university students commonly experience gender role stress derived from traditional masculine norms, yet there is a lack of targeted and acceptable psychological interventions for this population. High-intensity interval training, given its time efficiency and alignment with masculine values, may represent a potential strategy to improve resilience and alleviate gender role stress. This study aimed to examine the effects of an 8-week maximal power cycling intervention on resilience and gender role stress in male university students. Using a single-group pre-post design, 54 male university students were enrolled and underwent 8 weeks of Wingate sprint interval training (4 × 30-s all-out sprints, 4-min rest intervals, 2–3 sessions per week). Resilience (CD-RISC-10), masculine gender role stress (abbreviated MGRS), and rating of fatigue (ROF) were measured before and after the intervention. Paired *t*-tests, correlation analyses, and exploratory mediation analyses were conducted. Results showed that peak cycling power (*p* = 0.0007) and duration (*p* = 0.0018) significantly increased, resilience significantly improved (*p* = 0.0039), and gender role stress (*p* = 0.0150) and rating of fatigue (*p* < 0.001) significantly decreased. Exploratory mediation analysis revealed no significant indirect effect of resilience on the relationship between power improvement and gender role stress reduction (95% CI included 0). Of note, because all variables entered the model as change scores (Δ) in this single-group pre-post design, the temporal precedence requirement for formal mediation analysis could not be met; thus, these findings are exploratory and should not be interpreted as causal. Under this single-group pre-post design, significant improvements in resilience and reductions in gender role stress were observed after the 8-week intervention, but these changes warrant cautious interpretation. Due to the lack of a control group, the results should be considered preliminary and exploratory, and causality cannot be established. Moreover, the mechanism does not appear to operate through a mediating pathway of resilience, it may involve more complex psychological processes such as behavioral activation and cognitive restructuring of fatigue.

## 1 Introduction

Men have long been subjected to unique forms of gender role stress, which has contributed to serious mental health crises including elevated suicide rates and low help-seeking behaviors (Canetto and Sakinofsky, 1998; Rutz and Rihmer, 2009; Sher, 2020). This problem is equally salient among male university students, a specific subgroup of young men (Pitman et al., 2012). The gender role stress experienced by adolescent males stems from multiple factors, including the desire for success, competition for power, and the pursuit of self-dominance. Adherence to traditional masculinity norms may exacerbate their psychological conflicts and distress. Although prior research has identified the presence of masculine gender role stress among male university students, targeted psychological interventions to enhance resilience in this population remain scarce (Blazina et al., 2007; O’Neil et al., 1986; Wong et al., 2017). Historically, suicide risk has generally increased with age, with older adults once considered the highest-risk group. However, since the global rise in suicide cases toward the end of the 20th century, research attention has progressively shifted toward suicide rates among younger populations (Mann et al., 2005; Pitman et al., 2012). Studies have found that while suicide rates among older adults have declined, rates among younger cohorts have risen, and in most countries, suicide now ranks among the top three to five leading causes of death in young men (White and Holmes, 2006). Despite substantial overall declines in global suicide mortality, this issue remains unresolved, posing ongoing challenges for policy-making and health services that require further coordinated efforts to reduce suicide-related deaths (Turecki et al., 2019; Weaver et al., 2025).

Emerging evidence suggests that masculinity is a significant determinant of depression in men, and that traditional therapeutic approaches may not be broadly acceptable to this group, necessitating the development of alternative, masculinity-congruent interventions (Oliffe and Phillips, 2008). A substantial body of research indicates that physical activity, as a “male-friendly” intervention modality, may indirectly improve psychological outcomes through physical challenges. Exercise has been shown to exert positive effects on cognitive function, depression severity, sleep quality, alcohol consumption, and psychiatric symptoms (Czosnek et al., 2019; Martland et al., 2023). Moreover, exercise can attenuate sympathetic nervous system responses to stress (Silverman and Deuster, 2014), and multiple randomized controlled trials (RCTs) have confirmed that both aerobic and anaerobic exercise alleviate depressive symptoms and improve mental health (Stanton and Reaburn, 2014). Maximal cycling power training, which employs 30-s Wingate all-out sprints interspersed with rest intervals [adapted from the classic sprint interval training (SIT) protocol] (Burgomaster et al., 2005; Vollaard and Metcalfe, 2017), is classified as SIT—a high-intensity, short-duration form of training combining both anaerobic and aerobic components (Gibala et al., 2012). This format aligns with the time constraints and low exercise adherence commonly observed among university students (Gist et al., 2014; Godin et al., 1994).

Regarding the definition of resilience, a widely accepted conceptualization describes it as the capacity of a dynamic system to adapt successfully to disruptions that threaten its viability, function, or development (Masten, 2001; Masten, 2014). Resilience also encompasses hope and meaning. For individuals facing adversity, the sense of “meaning-making” is crucial; resilience fundamentally relies on the belief that life retains meaning, even in the face of chaos, cruelty, stress, worry, or despair (Panter-Brick and Eggerman, 2011; Southwick et al., 2014). Clough et al. (2002) proposed that mental toughness comprises four core psychological attributes: Confidence, Challenge, Commitment, and Control—that is, the ability to persist in challenging situations, maintain unwavering belief under pressure (including confidence in one’s abilities and interpersonal relationships), regulate emotions, and maintain control over one’s life (Clough et al., 2002). Furthermore, mental toughness is closely associated with stress management, stress recovery, and overall mental health (Gerber et al., 2012). In various human activities—including academic pursuits, sport, and work—mental toughness serves as an important prerequisite for consistent performance (McGeown et al., 2016). With respect to mental toughness profiles, athletes engaged in long-term sport participation typically exhibit higher levels of mental toughness. Mental toughness has been linked to multidimensional athlete characteristics (Clough et al., 2002; Cowden and Meyer-Weitz, 2016; Crust et al., 2014). From a gender perspective, multiple studies have confirmed that male athletes demonstrate higher mental toughness than their female counterparts, potentially attributable to greater confidence in physical (athletic) abilities and higher testosterone levels that may facilitate stronger mental resilience (Carney et al., 2010; Meggs et al., 2019). In addition, total mental toughness scores are significantly positively correlated with academic achievement and academic progression (Crust et al., 2014). It is important to clarify two concepts that are often confused: resilience and mental toughness. Resilience is generally defined as the successful adaptation of individuals to adversity, trauma, or significant stress, with its core emphasis on “bouncing back” and meaning-making capacities. Mental toughness, by contrast, places greater emphasis on trait-like characteristics of maintaining focus, confidence, and control under pressure, as exemplified by Clough et al. (2002) 4C model. Although overlapping to some extent, the two constructs originate from different theoretical traditions and are assessed by distinct measurement tools. The present study selected the CD-RISC-10 to measure resilience, based on the following considerations: (1) the CD-RISC-10 has established good reliability and validity in university student populations; (2) the study focused on adaptive changes following stress exposure, rather than stable trait performance in competitive contexts; and (3) its brevity makes it suitable for use in conjunction with high-intensity exercise interventions.

Against this background, the present study employed a single-group pre-post design to investigate the effects of an 8-week Wingate maximal power cycling training program on resilience and masculine gender role stress in male university students aged 18–22 years. The research hypotheses were as follows:

*H1*: After the 8-week maximal power cycling intervention, resilience (CD-RISC-10 scores) of male university students would significantly increase;

*H2*: The 8-week maximal power cycling intervention would significantly reduce masculine gender role stress (abbreviated MGRS scores) in male university students;

*H3*: Peak cycling power and duration would significantly improve after the intervention;

*H4* (exploratory): Resilience would mediate the relationship between power improvement and gender role stress reduction. It should be noted in advance that, due to the single-group pre-post design, all variables entered the model as pre-to-post change scores (Δ), which cannot satisfy the temporal precedence requirement for formal mediation analysis—that is, it cannot be determined that improvements in power and resilience preceded reductions in gender role stress in time. Therefore, the mediation analysis in this study is exploratory and data-driven; the results are intended only to describe covariation among variables and do not support any causal inferences.

## 2 Materials and methods

### 2.1 Participants

#### 2.1.1 Study design and ethical approval

This study employed a single-group pre-post design. The study protocol was approved by the Ethics Committee of Sichuan Agricultural University (approval number: H20250055; date of approval: November 12, 2025) and was conducted in strict accordance with the Declaration of Helsinki. All participants provided written informed consent voluntarily after being fully informed of the study purpose, procedures, potential risks, and benefits. Participants were entitled to withdraw at any time, unconditionally and without obligation, and all data from those who withdrew were removed in accordance with the protocol. This study was not prospectively registered, as it was designed as an exploratory pilot study with a single-group pre-post design, intended to generate preliminary evidence for future randomized controlled trials. All personal data were anonymized upon collection, with participant identities replaced by study codes. Original identifying information and data were stored separately on different password-protected computers, accessible only to the principal investigator. Data will be retained for 5 years for verification purposes and destroyed thereafter in accordance with relevant regulations. All data processing and analyses complied with the Declaration of Helsinki and the regulations of the Ethics Committee of Sichuan Agricultural University.

#### 2.1.2 Sample size estimation

Sample size was estimated a priori using G*Power 3.1 software. Assuming a paired-sample *t*-test (two-tailed), a medium effect size (Cohen’s *d* = 0.5), α = 0.05, and statistical power (1-β) = 0.80, the minimum required sample size was calculated to be 34 participants. To account for an anticipated dropout rate of approximately 20%, 60 participants were actually recruited. Ultimately, 54 participants completed the full intervention and all measurements, yielding a completion rate of 90.0%, which exceeded the pre-specified adherence criterion of 80%.

#### 2.1.3 Participant recruitment

Recruitment period: October 15, 2025 to November 1, 2025.

Recruitment methods: Recruitment announcements were disseminated through multiple channels, including campus bulletin boards at Sichuan Agricultural University, college-level WeChat groups, class QQ groups, and in-*class oral presentations. The recruitment information included the study purpose, intervention content, time commitment (8 weeks, 2–3 training sessions per week), inclusion and exclusion criteria, and contact information of the research team. Interested individuals registered by scanning a QR code or contacting a research assistant.

Screening procedure: A total of 127 applications were received. Research assistants first conducted an initial screening via telephone or online questionnaire, excluding 42 individuals who did not meet the inclusion criteria. The remaining 85 candidates were invited to attend in-person screening interviews, during which the principal investigator performed detailed verification of inclusion/exclusion criteria, including: explaining the study procedures in detail and confirming participants’ understanding and acceptance of the time commitment; collecting health history and exercise history information; and administering the Physical Activity Readiness Questionnaire (PAR-Q).

Following the screening interviews, 25 individuals were excluded [reasons: time conflicts preventing full participation (*n* = 14), PAR-Q positivity requiring further medical evaluation (*n* = 6), and concerns about training intensity (*n* = 5)]. A total of 60 eligible participants were ultimately enrolled.

#### 2.1.4 Inclusion and exclusion criteria

Inclusion criteria:

(1) Male university students aged 18 ultyears;
(2) No systematic regular exercise training in the previous 3 months (systematic training was defined as participation in moderate-to-vigorous intensity exercise ≥ 2 sessions per week, ≥ 30 min per session);
(3) Good physical health, free from cardiovascular disease, metabolic disorders, or other chronic conditions that might affect exercise participation;
(4) Willing to participate voluntarily and to provide written informed consent.

Exclusion criteria:

(1) History of musculoskeletal injury (e.g., fractures, ligament tears) that had not fully recovered;
(2) Diagnosis of a psychiatric disorder (e.g., depression, anxiety, schizophrenia) or receipt of professional treatment for mental health issues within the past year;
(3) Substance dependence or alcohol abuse;
(4) Answered “yes” to any item on the PAR-Q and deemed unsuitable for high-intensity exercise following evaluation by a study physician;
(5) Concurrent participation in another interventional study.

#### 2.1.5 Participant flow and attrition

**FIGURE 1:**
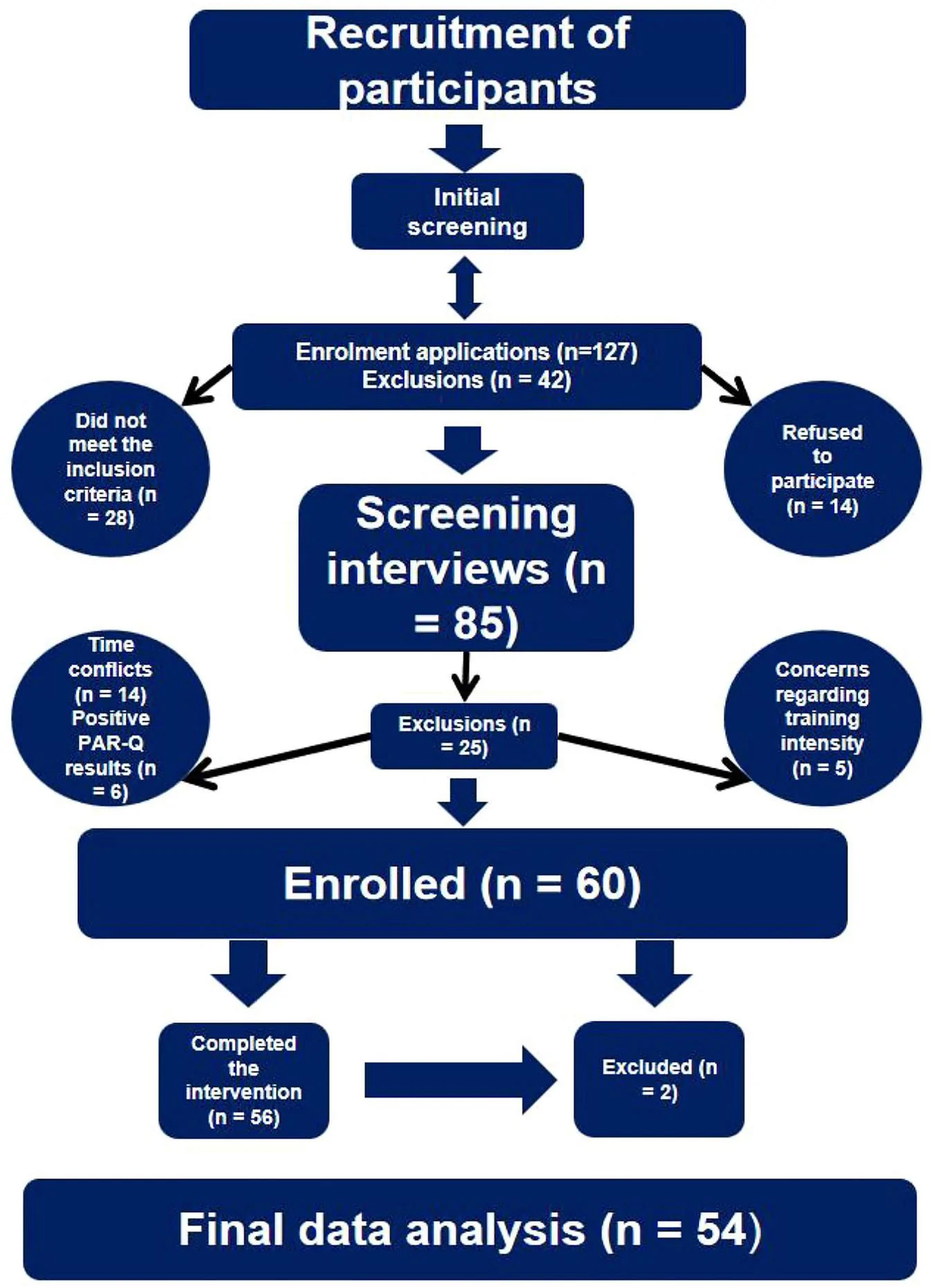
Participant recruitment process.

The recruitment and allocation procedures of the study participants are presented in Figure 1.

Description of attrition: Among the 60 enrolled participants, 56 completed the full 8-week intervention (completion rate: 93.3%), and 54 were included in the final data analysis. Six participants were excluded or withdrew for the following reasons: acute ankle sprain (unrelated to training) (*n* = 1), upper respiratory tract infection (*n* = 1), upper respiratory tract infection (*n* = 1), personal reasons (*n* = 1), and failure to complete ≥ 80% of training sessions (completing 13 and 15 sessions, respectively) (*n* = 2).

#### 2.1.6 Informed consent and data privacy

All participants signed written informed consent forms prior to the start of the study. The consent forms detailed the study purpose, training schedule, potential risks (including muscle soreness, fatigue, etc.), anticipated benefits, and the right to withdraw. Participants were informed that their personal data would be anonymized, used solely for the purposes of this study, and stored on password-protected computers. Data will be retained for 5 years after study completion for verification purposes.

### 2.2 Single-group pre-post intervention design

All 60 male university participants (*n* = 60) were assigned to the experimental group, with no control group included. The intervention consisted of 8 weeks of maximal power training on a Wingate cycle ergometer (Monark 824E, Sweden), comprising 4 × 30-s sprints with 4-min rest intervals, conducted 2–3 times per week [Cd = 0.075; cycle ergometer resistance = body weight (kg) × Cd]. Prior to each training session, participants performed a 5-min warm-up at 60 rpm with zero resistance to activate the neuromuscular system and acclimatize to the testing environment. Following the warm-up, a low-intensity active recovery period was conducted at a constant workload of 50 W (Arazi et al., 2015; Bao et al., 2022). The 8-week intervention adopted a progressive loading design, consisting of two phases: an initial adaptation phase (weeks 1–3: 4 × 30-s sprints, 4-min intervals, 2 sessions/week) and an intensification phase (weeks 4–8: 4 × 30-s sprints, 4-min intervals, 3 sessions/week). The adaptation phase was designed to gradually familiarize participants with the training intensity, improve training quality, and minimize adverse responses, whereas the intensification phase aimed to further challenge participants and enhance training stimulus. Upon completion of the entire intervention period, changes in peak power output (PPO) and its duration were compared, alongside the administration of stress and fatigue (ROF) scales, to explore the association between pain tolerance during high-intensity exercise and the alleviation of masculine gender role stress in male university students.

### 2.3 Methods

#### 2.3.1 Time and location

(1) Time: 12 November 2025 to 6 January 2026, a total of 8 weeks.
(2) Location: Ya’an Key Laboratory of Sports Human Science and National Physical Fitness Health Promotion.

#### 2.3.2 Measurement tools

Primary outcome: The 10-item Connor-Davidson Resilience Scale (CD-RISC-10). This study employed the Chinese version of the CD-RISC-10, originally developed by Campbell-Sills and Stein (2007) and subsequently adapted by Campbell-Sills and Stein (2007). The scale consists of 10 items rated on a 5-point Likert scale (1 = “not true at all” to 5 = “true nearly all the time”), with higher scores indicating higher levels of resilience. Although the present sample comprised general undergraduate male students, which differs somewhat from the vocational college student population in terms of educational background, the psychometric validation conducted by Guan et al. (2022) among 1,175 vocational college students demonstrated that the CD-RISC-10 has a Cronbach’s α coefficient of 0.952, a Spearman-Brown split-half reliability coefficient of 0.936, and a 3-week test-retest reliability of 0.75. Exploratory factor analysis extracted a single factor accounting for 70.431% of the cumulative variance, with factor loadings for individual items ranging from 0.797 to 0.870. Confirmatory factor analysis indicated good model fit after modification (χ^2^/df = 4.699, GFI = 0.950, CFI = 0.974, RMSEA = 0.079). The total score of the scale was significantly correlated with the K-10 (*r* = −0.634), GHQ-12 (*r* = −0.496), and WEMWBS (*r* = 0.846), all at *p* < 0.001, suggesting acceptable construct and criterion validity of the scale in Chinese student populations. Based on the above psychometric evidence, the Chinese version of the CD-RISC-10 is considered suitable for assessing resilience among Chinese university students. In the present sample, the Cronbach’s α coefficients were 0.89 (pre-test) and 0.91 (post-test), indicating good internal consistency (Guan et al., 2022).

Secondary outcome: Masculine gender role stress was assessed using the Masculine Gender Role Stress Scale (MGRS). One of the most widely used assessment tools is the MGRS developed by Eisler and Skidmore (1987) (Eisler and Skidmore, 1987), which comprises 40 items rating stress responses across various situations. However, due to its length and potential inefficiency, the present study adopted the abbreviated version of the MGRS (Swartout et al., 2015), which includes 15 items to reduce participant burden and assessment costs. Items are rated on a 6-point Likert scale (0 = “not at all” to 5 = “extremely”).

Behavioral task: This study employed the Rating of Fatigue (ROF) scale (Micklewright et al., 2017) to track perceived fatigue across daily life, physical activity, and recovery contexts. Prior to its use, participants received standardized training on the concept and levels of fatigue to ensure accurate ratings. The ROF is an 11-point numerical rating scale on which participants provide a single-number response from 0 (“no fatigue”) to 10 (“exhausted”), responding intuitively and without hesitation. In this study, ROF ratings were obtained immediately following each Wingate test session, for both pre-test and post-test. The limitations arising from this procedure are addressed in the Discussion.

### 2.4 Data analysis

A total of 54 participants (*n* = 54) were included in the final data analysis. All statistical analyses were performed using Python (including paired-samples *t*-tests and Pearson correlation analyses). Because the pre-intervention ROF data showed no variability (SD = 0.00), the Wilcoxon signed-rank test was additionally employed as a robust alternative.

The statistical analyses were categorized into confirmatory and exploratory levels. For the paired-samples *t*-tests (Table 1), which involved four primary outcome indicators and one manipulation check, the Bonferroni method was applied to control for Type I error inflation. The corrected significance threshold was set at α = 0.05/5 = 0.01 (two-tailed) (Bland and Altman, 1995). Correlation analyses (Table 2) and mediation analyses (Tables 3, 4) were considered exploratory and were not subjected to multiple comparison correction; their results are reported as exploratory findings, and all *p*-values are provided for descriptive reference only, not as the basis for definitive conclusions. Interpretations of these exploratory analyses have taken this limitation into account.

**TABLE 1:** Paired-sample *t*-test results for each indicator before and after intervention (*n* = 54).

Indicator	Pre-intervention (M ± SD)	Post-intervention (M ± SD)	*t*	*p*	Result
High-power cycling power (w)	135.37 ± 16.25	152.91 ± 14.78	3.70	0.0007	Significant increase
High-power cycling duration (s)	13.32 ± 2.14	15.42 ± 2.24	3.30	0.0018	Significant prolongation
Gender role stress	29.85 ± 1.01	29.22 ± 1.00	−2.52	0.0150	Significant decrease
Resilience (CD-RISC-10)	34.46 ± 1.12	35.06 ± 1.21	3.00	0.0039	Significant increase
Fatigue perception (manipulation check)	10.00 ± 0.00	8.30 ± 0.48	—	<0.001	Significant decrease

The ROF metric is provided as Supplementary Information and is not considered a primary outcome; it is used solely for manipulation checks.

**TABLE 2:** Changes, effect sizes, and 95% confidence intervals for primary outcomes from pre- to post-intervention (*n* = 54).

Indicator	Pre-intervention (M ± SD)	Post-intervention (M ± SD)	95% CI for Δ	Effect size	*p*
High-power cycling power (w)	135.37 ± 16.25	152.91 ± 14.78	[8.03, 27.05]	0.503	0.0007
High-power cycling duration (s)	13.32 ± 2.14	15.42 ± 2.24	[0.83, 3.37]	0.449	0.0018
Gender role stress	29.85 ± 1.01	29.22 ± 1.00	[−1.13, −0.13]	−0.343	0.0150
Resilience (CD-RISC-10)	34.46 ± 1.12	35.06 ± 1.21	[0.20, 1.00]	0.408	0.0039
Fatigue perception^†^	10.00 ± 0.00	8.30 ± 0.48	[−1.83, −1.57]	0.866^‡^ (r)	<0.001

† ROF data at baseline showed no variability (SD = 0.00), violating the normality assumption for paired *t*-test. The Wilcoxon signed-rank test was used instead (*Z* = −6.364, *p* < 0.001). ^‡^ For ROF, the effect size is reported as *r* = | Z| / √n (non-parametric analog), which indicates a large effect. The 95% CI for Δ is shown for descriptive purposes only. Effect size interpretation: For Cohen’s d < sub > z < /sub > (or r), values of 0.2, 0.5, and 0.8 correspond to small, medium, and large effects, respectively.

**TABLE 3:** Pearson correlation coefficients and significance of change scores (*n* = 54).

Variable	Δ Power	Δ Gender role stress	Δ Mental toughness	Δ Fatigue
ΔPower	1.000	−	−	−
ΔGender role stress	0.096 [−0.178, 0.370] (*p* = 0.492)	1.000	−	−
ΔResilience (CD-RISC-10)	0.013 [−0.261, 0.287] (*p* = 0.925)	0.429 [0.185, 0.637] (*p* = 0.001)	1.000	−
ΔFatigue	−0.593 [−0.958, −0.410] (*p*<0.001)	−0.333 [−0.620, −0.072] (*p* = 0.014)	0.048 [−0.226, 0.322] (*p* = 0.731)	1.000

Each cell in the table is formatted as “correlation coefficient r/[95% confidence interval]/*p*-value.” All confidence intervals are calculated based on the Fisher Z-transformation. As the analysis in this section is exploratory, no correction for multiple comparisons has been applied; this limitation has been taken into account in the interpretation of the results.

**TABLE 4:** Path coefficients and test results for the mediation effect of mental toughness.

Path	Path coefficient	*p*	95% Bootstrap CI
Total effect c (X→Y)	0.0046	0.7926	−
Path a (X→M)	0.0009	0.9720	−
Path b (M→Y)	0.3053	0.2488	−
Direct effect c’ (X→Y, controlling for M)	0.0044	0.7983	−
Indirect effect (a × b)	0.0003	−	[−0.0582, 0.0162]

It should be particularly noted that all mediating and outcome variables in this study entered the model as pre-to-post change scores (Δ), i.e., Δ power →Δ resilience →Δ gender role stress. This analytical approach can only reflect covariation among variables and cannot satisfy the temporal precedence requirement for formal mediation analysis—that is, it cannot be established whether improvements in power and resilience temporally preceded reductions in gender role stress. Therefore, these analyses are strictly defined as “exploratory covariation analyses based on change scores,” and their results should not serve as the basis for any causal inference; they are interpreted only in an exploratory manner within the Discussion.

## 3 Results

### 3.1 Pre-post paired-sample *t*-tests for each outcome indicator

**FIGURE 2:**
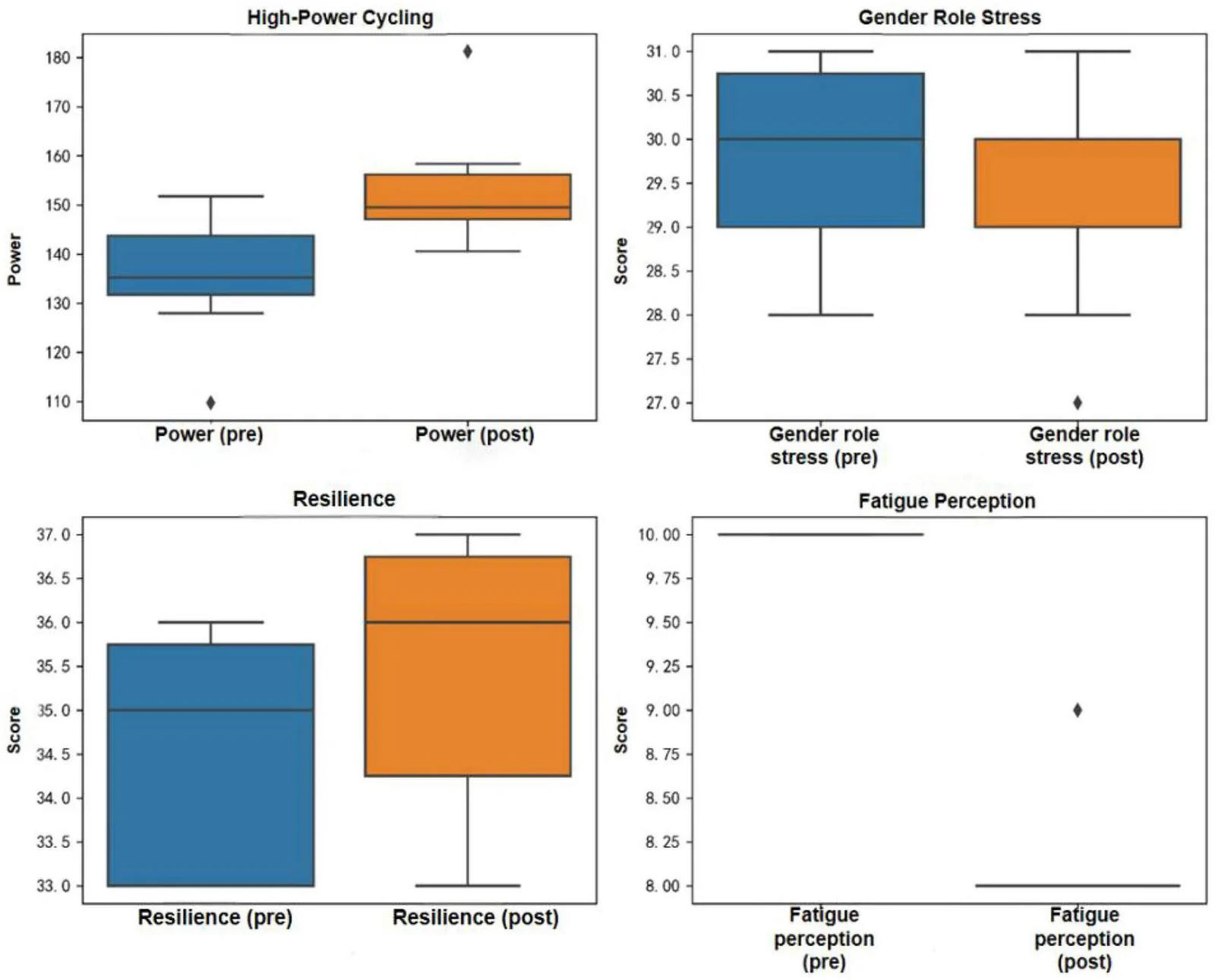
Boxplots of each indicator before and after intervention.

Prior to hypothesis testing, normality of the pre-to-post change scores (Δ) for each indicator was examined using the Shapiro–Wilk test, and outliers were screened using the boxplot method [1.5 × interquartile range (IQR) criterion]. The results showed that the change scores for peak power output (*W* = 0.976, *p* = 0.312), time to peak power (*W* = 0.981, *p* = 0.451), gender role stress (*W* = 0.972, *p* = 0.218), and resilience (*W* = 0.968, *p* = 0.162) all conformed to normal distributions (*p* > 0.05), and no extreme outliers beyond 1.5 × IQR were detected. Accordingly, paired-sample *t*-tests were used for pre-post comparisons of these four indicators. For rating of fatigue (ROF), because the pre-intervention data showed no variability (SD = 0.00), the Wilcoxon signed-rank test was employed as the primary analytical method; results are presented in Table 1. The Bonferroni method was applied to control for multiple comparisons across the five primary outcome indicators and Figure 2, with the corrected significance threshold set at α = 0.01 (two-tailed). The results showed that peak power output (*p* = 0.0007) and time to peak power (*p* = 0.0018) significantly increased after the intervention; resilience (*p* = 0.0039) significantly improved; and rating of fatigue (*p* < 0.001) significantly decreased. Although gender role stress decreased after the intervention (*p* = 0.0150), this difference did not reach the adjusted significance level after Bonferroni correction (α = 0.01), indicating that this change warrants cautious interpretation. Detailed change scores, effect sizes, and 95% confidence intervals for each indicator are presented in Table 2.

For the ROF indicator, given that all participants scored 10.00 (SD = 0.00) at pre-intervention, indicating a pronounced ceiling effect, the Wilcoxon signed-rank test was additionally conducted. The results showed that the pre-post difference remained significant (*Z* = −6.364, *p* < 0.001), consistent with the conclusions drawn from the paired-sample *t*-test.

Beyond statistical significance, this study further calculated effect sizes and 95% confidence intervals for the change scores to evaluate the practical significance of the observed changes. As shown in Table 2, the effect size for peak power output was moderate (d_z = 0.503), with a mean increase of 17.54 W [95% CI: (8.03, 27.05)]; the effect size for resilience was small to moderate (d_z = 0.408), with a mean increase of 0.60 points [95% CI: (0.20, 1.00)]; the effect size for gender role stress was small to moderate (d_z = −0.343), with a mean decrease of 0.63 points [95% CI: (−1.13, −0.13)]; and the effect size for rating of fatigue was large (*r* = 0.866), with a mean decrease of 1.70 points [95% CI: (−1.83, −1.57)]. Overall, with the exception of fatigue, the effect sizes for the primary outcome indicators ranged from small to moderate, suggesting that although statistically significant, the magnitudes of change were limited in practical terms.

### 3.2 Pearson correlation analysis of change scores

**FIGURE 3:**
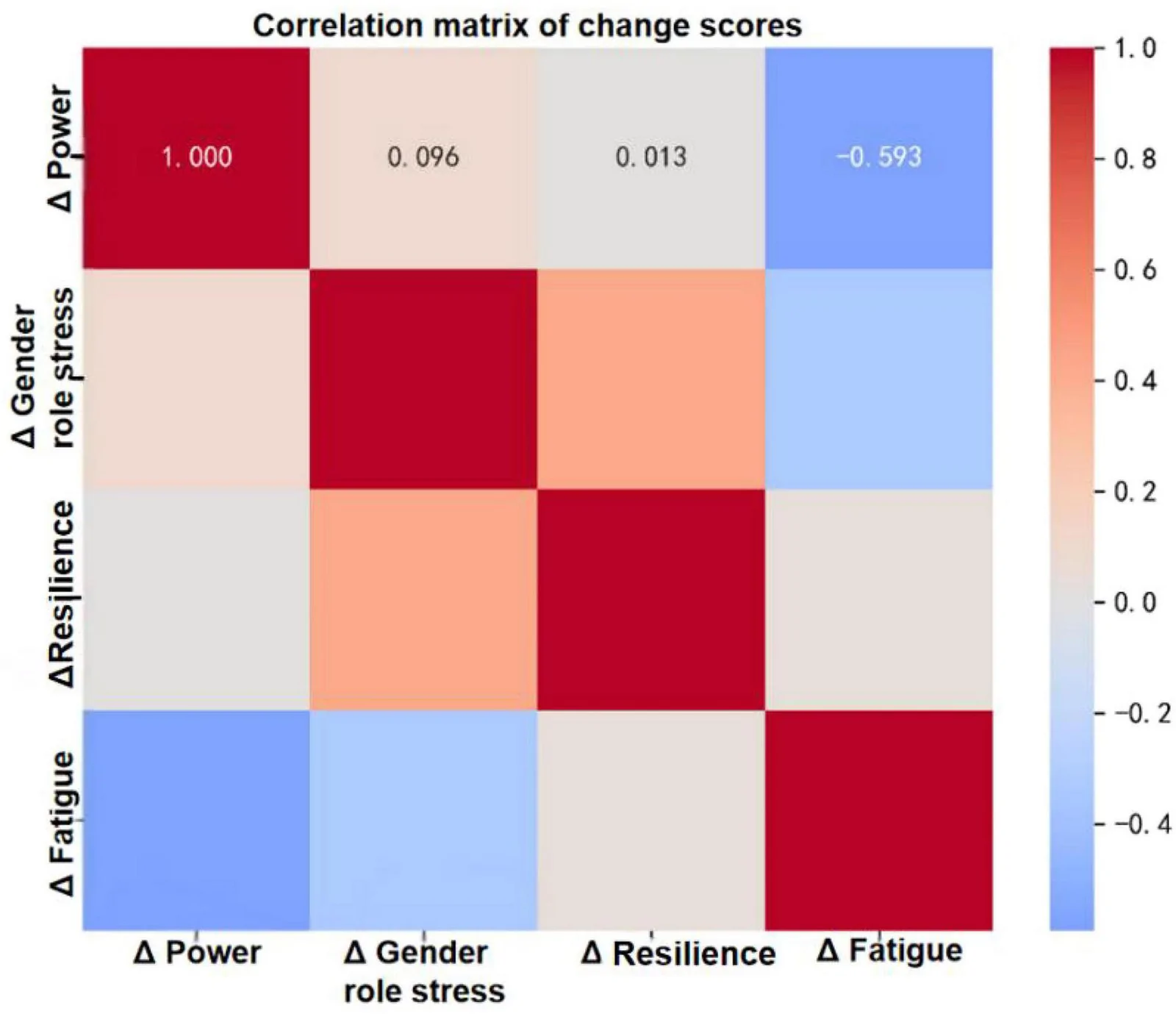
Correlation matrix of change scores for each indicator.

Pearson correlation coefficients were calculated for Δ power, Δ gender role stress, Δ resilience, and Δ fatigue. It should be noted that significance tests for the correlations in this section were not adjusted for multiple comparisons, and all *p*-values are provided for descriptive reference only. Detailed data are presented in Table 3 and Figure 3, with Figure 3 displaying the correlation matrix of change scores for all indicators as a heatmap. The results showed a significant negative correlation between Δ power and Δ fatigue (*r* = −0.593, *p* < 0.001), indicating that improvements in power were closely associated with reductions in fatigue. Notable significant relationships were also observed between Δ gender role stress and Δ resilience (positive correlation, *r* = 0.429) and between Δ gender role stress and Δ fatigue (negative correlation, *r* = −0.333), both reaching statistical significance (*p* = 0.001 and *p* = 0.014, respectively). Non-significant relationships included those between Δ power and Δ gender role stress, Δ power and Δ resilience, and Δ resilience and Δ fatigue, with *p*-values all exceeding 0.05 and confidence intervals crossing zero.

### 3.3 Exploratory mediation analysis of mental toughness

Based on the limitations described above (i.e., the inability to satisfy the temporal precedence assumption), this section is presented as an exploratory covariation analysis rather than a formal mediation test. A path model was constructed with Δ power (X) →Δ resilience (M) →Δ gender role stress (Y). The results showed that none of the path coefficients reached statistical significance, and the 95% bootstrap confidence interval for the indirect effect crossed zero, indicating no evidence of a significant indirect covariation of resilience in the relationship between power improvement and gender role stress reduction. Detailed data are presented in Table 4. Because the temporal precedence assumption was not met, this result should not be interpreted as “resilience does not mediate the relationship,” but rather as “no significant indirect covariation was detected under the present study design.”

The total effect was tested using the bootstrap method with 5,000 resamples. The results showed that the total effect (X → Y) was not significant (path coefficient = 0.0046, *p* = 0.7926); path a (X → M) was not significant (coefficient = 0.0009, *p* = 0.9720); path b (M → Y) was not significant (coefficient = 0.3053, *p* = 0.2488); and the direct effect c’ (X → Y, controlling for M) was not significant (coefficient = 0.0044, *p* = 0.7983). The estimated indirect effect (a × b) was 0.0003, with a 95% bootstrap confidence interval of [−0.0582, 0.0162], which included zero, indicating non-significance. Given that the total effect was not significant, the mediation model did not meet the prerequisites for standard mediation analysis, and the results are reported only as exploratory findings.

### 3.4 Exploratory parallel mediation analysis of mental toughness and fatigue

Also due to the temporal precedence limitation, this section is presented as an exploratory parallel covariation analysis. No correction for multiple comparisons was applied, and all *p*-values are provided for descriptive reference only. Resilience and fatigue were entered as parallel variables into the path model. The regression analysis showed that neither variable significantly predicted Δ gender role stress; the 95% bootstrap confidence intervals for both indirect effects crossed zero, indicating no evidence of significant indirect covariation. Detailed data are presented in Table 5.

**TABLE 5:** Regression coefficients and indirect effect results for parallel mediation.

Variable	Regression coefficient (B)	Standard error (SE)	*t*	*p*	Indirect effect	95% CI
Constant	−2.1069	1.187	−1.774	0.126	−	−
ΔPower	−0.0090	0.020	−0.448	0.670	−	−
ΔMental toughness	0.3237	0.239	1.354	0.224	−0.0081	[−0.1165, 0.0170]
ΔFatigue	−0.7756	0.693	−1.120	0.306	0.0065	[−0.0305, 0.0380]

Specifically, the constant term was not significant (*B* = −2.1069, SE = 1.187, *t* = −1.774, *p* = 0.126); the regression coefficient for Δ power was not significant (*B* = −0.0090, SE = 0.020, *t* = −0.448, *p* = 0.670); the coefficient for Δ resilience was not significant (*B* = 0.3237, SE = 0.239, *t* = 1.354, *p* = 0.224); and the coefficient for Δ fatigue was also not significant (*B* = −0.7756, SE = 0.693, *t* = −1.120, *p* = 0.306). Furthermore, the indirect effect of Δ resilience was −0.0081 [95% CI (−0.1165, 0.0170)], and the indirect effect of Δ fatigue was 0.0065 [95% CI (−0.0305, 0.0380)]; both confidence intervals included zero, indicating that the total effect was not significant. As with the previous analysis, this result should not be interpreted as definitive evidence against parallel mediation, but rather as an indication that no significant indirect covariation was detected in this exploratory analysis.

## 4 Discussion

In this 8-week Wingate sprint interval training program, significant improvements were observed in participants’ peak power output and the duration of high-power maintenance. This finding is consistent with multiple studies on the physiological mechanisms of high-intensity interval training. Burgomaster et al. (2005) reported that as few as six sessions of 30-s all-out sprint training significantly enhanced muscle oxidative capacity and cycling endurance (Burgomaster et al., 2005). Subsequent research further indicated that low-volume, high-intensity interval training can rapidly induce muscle metabolic adaptations, including enhanced mitochondrial biogenesis and increased glycogenolytic enzyme activity, thereby enabling higher power output within a short period (Gibala et al., 2006; Gibala et al., 2012; Parolin et al., 1999). There is now a general consensus regarding the efficacy of high-intensity interval training in improving both anaerobic and aerobic capacity, and research attention has gradually shifted toward dose-response relationships within training protocols. Evidence suggests that SIT protocols with shorter sprint durations and fewer repetitions can still significantly improve maximal oxygen uptake (VO_2_max) and anaerobic power (Vollaard and Metcalfe, 2017); reducing the work interval by 50% does not diminish aerobic adaptations and does not affect increases in lactate threshold or critical power (Zelt et al., 2014). In addition, a study conducted with male university students demonstrated that reducing the number of sprints in the classic SIT protocol and incorporating more active recovery similarly enhanced anaerobic capacity, further validating the effectiveness of optimized protocols (Huang et al., 2025). Notably, the progressive loading design adopted in the present study (2 sessions/week during the adaptation phase and 3 sessions/week during the intensification phase) may have played a key role in optimizing training adaptation. Previous evidence suggests that such a design helps alleviate initial muscle soreness and psychological resistance commonly experienced during the early stages of high-intensity training, thereby improving long-term adherence—a factor of particular importance in university student populations (Godin et al., 1994). Furthermore, the resistance setting of the cycle ergometer in this study was body-weight-dependent (Cd = 0.075), and such individualized loading may have further facilitated effective improvements in power output (Laursen et al., 2002). Future research could incorporate muscle biopsy and blood lactate kinetic analyses to further elucidate the metabolic and molecular mechanisms underlying power enhancement.

The significant improvement in the experimental group’s CD-RISC-10 scores following the intervention supports the theoretical hypothesis that “high-intensity physical challenges can enhance resilience.” From the perspective of resilience as a dynamic developmental process, this finding can be understood through the lens of stress inoculation theory. The exercise modality in this study—all-out sprints interspersed with recovery intervals—essentially constituted a form of structured stress exposure. Through repeated experiences of the “physiological limit–recovery–rechallenge” cycle, individuals may develop more adaptive stress appraisal and emotion regulation strategies. This aligns with the core logic of stress inoculation training, which posits that through controlled, gradual exposure to stressors, individuals can progressively develop more effective coping skills and cognitive schemas (Meichenbaum, 2007). In this study, high-intensity physiological stress served as the “inoculation” material, with each recovery period providing an opportunity for cognitive integration and physiological adaptation, ultimately enhancing overall adaptive capacity—a process that is highly consistent with the core essence of resilience. This interpretation is further supported by posttraumatic growth theory, which suggests that highly resilient individuals not only “get through” adversity but may also discover new meaning, establish new relationships, or gain personal strength, thereby achieving growth (Masten, 2001; Tedeschi and Calhoun, 2004). Furthermore, the resilience traits measured by the CD-RISC-10 have been shown to be enhanced through behavioral interventions (Campbell-Sills and Stein, 2007), and exercise represents an effective behavioral activation strategy. Large-scale studies have confirmed that physical activity is significantly negatively associated with depressive symptoms, supporting the incorporation of exercise as a key component of “behavioral activation interventions” in public health and clinical settings (Stubbs et al., 2016). Future research could adopt mixed-method designs, incorporating training logs and semi-structured interviews, to more deeply explore participants’ subjective experiences of “resilience moments” during the training process.

Using the abbreviated Masculine Gender Role Stress Scale, this study found that gender role stress scores decreased after the intervention (*p* = 0.015). However, it should be noted that after applying Bonferroni correction for multiple comparisons across the four primary outcome indicators (α = 0.01), this difference did not reach the adjusted statistical significance threshold, indicating that this change should be cautiously interpreted as a trend-level finding rather than a definitive conclusion. When exploring this trend-level finding from an exploratory perspective, existing theories suggest that excessive conformity to traditional masculine norms is significantly associated with depression, anxiety, and role conflict (Wong et al., 2017), and that men often avoid traditional psychological treatment due to stigma, yet are more receptive to intervention modalities delivered through physical challenges (Oliffe and Phillips, 2008). High-intensity cycling, as a “male-friendly” activity aligned with masculine socialization expectations, may provide opportunities for stress release and self-efficacy enhancement without triggering gender role threat (Kimmel, 2013). The risk-taking and pain tolerance inherent in high-intensity cycling can be understood as an active coping response to the fear of being perceived as weak, potentially offering stress release, self-efficacy enhancement, and emotional regulation opportunities without activating gender role threat. Male gender role stress often originates from “fear of failure” and “pressure for emotional control,” and goal achievement in exercise (e.g., power improvement) along with a sense of physical mastery may directly or indirectly alleviate these core stressors (Biddle and Mutrie, 2007; Eisler and Skidmore, 1987). In addition, the “need to belong” theory suggests that a chronic lack of stable, positive interpersonal connections can lead to various negative psychological consequences, including intense feelings of isolation. The subtle presence of “benign competition” and “shared challenges” within the group training environment may attenuate feelings of isolation and facilitate flexible restructuring of gender role identity (Baumeister and Leary, 2017; Yu et al., 2026). Future research could incorporate the Gender Role Conflict Scale and measures of masculine ideology to more finely delineate the mediating pathways through which exercise interventions influence gender role stress.

Although high-intensity interval training induces acute fatigue, participants in this study showed a significant reduction in their rating-of-fatigue scores following the intervention, suggesting adaptive changes in individual perceptions and tolerance of fatigue. From a neuroendocrine perspective, regular exercise can enhance sympathetic nervous system adaptability to stress, reducing cortisol reactivity and thereby improving the efficiency of physiological stress recovery (Silverman and Deuster, 2014). The ROF scale emphasizes that fatigue perception is an integrated product of physiological and psychological factors, modulated by individual expectations, motivation, and emotional states (Micklewright et al., 2017). During the training process, participants may undergo cognitive reappraisal of the meaning of fatigue: shifting from initially perceiving fatigue as a “pain signal” to later interpreting it as “evidence of effort” or “a marker of progress.” Cognitive appraisal theory (Lazarus and Folkman, 1984) provides a core framework for understanding this phenomenon—onovides a core framework for understanding this phenomeognitivindividual’s primary appraisal of whether the event is a “threat” or a “challenge,” and their secondary appraisal of “what can I do about it.” Reappraising fatigue from “threat” to “challenge” represents a classic example of cognitive restructuring, closely aligned with the “meaning-making” process emphasized in resilience research (Panter-Brick and Eggerman, 2011; Southwick et al., 2014). When participants redefined the fatigue experienced during high-intensity cycling from a “threatening signal” to “evidence of effort and progress,” they effectively accomplished an important cognitive reappraisal, which is a typical manifestation of the dynamic adaptive process of resilience. Previous evidence has shown that exercise interventions can improve fatigue symptoms in depressed individuals, partly attributable to improved sleep quality and enhanced self-efficacy (Stanton and Reaburn, 2014). Additionally, the structured recovery intervals (4 min) in this study may have taught participants how to actively manage energy allocation within stress cycles, thereby enhancing overall fatigue regulation capacity. This is reflected in the fact that high-intensity interval training not only improves physiological parameters such as VO*2*max but also enhances “exercise economy” and “endurance” (Laursen and Jenkins, 2002). Such improvements arise in part from neuromuscular coordination and psychological adaptation, the latter including a more effective capacity to tolerate and manage repetitive high-intensity efforts repetitive highsychological to energy allocation skills. However, a notable phenomenon deserving special attention in this study is that the mean pre-intervention ROF score was 10.00 (SD = 0.00), meaning that all 54 participants reported the maximum score of “exhausted” prior to the intervention. This suggests that the ROF scale exhibited a ceiling effect in the present sample. Possible reasons include: first, the pre-intervention ROF measurement was conducted immediately after the high-intensity cycling test, at which point participants were at the peak of acute physiological fatigue, making the “exhausted” report physiologically plausible. Second, the SD = 0.00 indicates no variability among all participants, which does not exclude the possibility that some participants may have misinterpreted “fatigue” as “effort willingness” or “exertion during exercise” rather than actual perceived fatigue. Future research should provide standardized training on the concept of fatigue prior to measurement and consider using multidimensional fatigue assessment tools (e.g., the Multidimensional Fatigue Inventory, MFI-20) to enhance measurement sensitivity.

The exploratory covariation analysis in this study showed that in the path model of Δ power →Δ resilience →Δ gender role stress, none of the path coefficients reached statistical significance, and the 95% bootstrap confidence interval for the indirect effect included zero. Under the present study design, no significant indirect covariation evidence for resilience was detected. Taking the above results as exploratory findings, several speculative interpretations may be considered.

First, the emergence of covariation among variables may require a longer intervention period or a more intensive training frequency to stabilize. A large-sample study involving 1,091 adolescents reported a similar pattern: physical exercise significantly predicted resilience, but resilience did not play an independent mediating role between exercise and behavioral outcomes; rather, ego depletion showed a significant independent mediating effect in that pathway, and resilience and ego depletion together formed a chain mediation (Gan et al., 2025). This suggests that the effects of physical exercise on psychological outcomes may not operate through a single psychological trait pathway, but may involve more complex multivariate interactive processes.

Second, resilience may not function directly as a “mediator,” but rather as a psychological resource that operates more as a “moderator” or “contextual variable.” Some scholars have suggested that what truly enables individuals with high resilience to perform well under stressful conditions may not be resilience itself, but rather the coexisting psychological constructs such as emotional intelligence, self-efficacy, and motivation (Gameiro et al., 2023; Nicholls et al., 2015). In other words, resilience is more akin to an “outcome indicator” (reflecting an individual’s comprehensive adaptive capacity in the face of adversity) than a “mechanism variable” that explains causal relationships among other variables.

Third, the potential effects of high-intensity exercise on male gender role stress may depend more on behavioral activation, enhanced physical mastery, and satisfaction of belongingness needs within group training, rather than solely through a mediating pathway of resilience. Previous research has examined the mediating role of the behavioral activation system between physical activity level and depressive symptoms, finding that behavioral activation exerted a significant mediating effect in that pathway, suggesting that exercise may improve psychological states by activating the behavioral system, increasing positive reinforcement, and reducing avoidance behaviors (Zhu et al., 2023). The “mastery hypothesis” also provides supporting explanatory power, proposing that successfully completing a task requiring effort generates a sense of mastery, which in turn improves psychological states (Miller, 2004). The goals in high-intensity cycling (e.g., power improvement, sprint completion) are specific and provide immediate feedback, aligning with male psychological needs for “achievement orientation” and potentially having more direct pathways to core dimensions of gender role stress (e.g., fear of failure). It should be reiterated that all the above interpretations are based on theoretical speculation, and the data from this study alone cannot provide empirical support for any causal direction or mediating mechanism. Future research should incorporate a broader range of psychological variables (e.g., self-efficacy, emotion regulation capacity, perceived social support) and adopt multi-time-point measurement designs to more comprehensively elucidate the psychological mechanisms through which high-intensity exercise influences male gender role stress.

The most prominent methodological limitation of this study concerns the temporal precedence issue in mediation analysis. As noted above, all variables entered the path model as pre-to-post change scores (Δ), and this analytical strategy can only reflect covariation among variables, failing to satisfy the core requirement of temporal precedence in traditional mediation analysis—that is, the exposure (power improvement) and mediator (change in resilience) must precede the outcome (change in gender role stress) in time. Because data were collected at only two time points (pre- and post-intervention), the sequence of changes among variables could not be determined. Therefore, all mediation results should be regarded as “exploratory covariation analyses based on change scores” rather than formal mediation tests. Future research should employ multi-time-point measurement designs (e.g., mid-intervention assessments, post-intervention follow-ups) or more rigorous analytical approaches such as cross-lagged panel models to verify potential temporal relationships among variables. In addition, another limitation in the statistical analysis is the issue of multiple comparisons. Although Bonferroni correction was applied to the five primary paired *t*-tests to control the familywise error rate, the correlation analyses and mediation analyses were exploratory and were not corrected for multiple comparisons. This decision was based on the following considerations: (1) these analyses were predefined as exploratory, and their results should be viewed as hypothesis-generating rather than hypothesis-testing; and (2) over-correction might increase the risk of Type II error, potentially masking meaningful exploratory findings. Nevertheless, readers should exercise caution when interpreting *p*-values from exploratory analyses, and results approaching the nominal significance level of 0.05 (e.g., gender role stress) should be regarded as trend-level evidence rather than definitive conclusions.

## 5 Conclusion

This study employed a single-group pre-post design, and the results should be regarded as exploratory preliminary evidence. Eight weeks of maximal power cycling training significantly improved peak power output, resilience, and subjective fatigue perception (the latter remained significant after Bonferroni correction) in male university students; although gender role stress decreased following the intervention, it did not reach the adjusted significance threshold after correction for multiple comparisons, representing a trend-level finding. Exploratory covariation analysis did not detect a significant indirect effect of resilience in the relationship between power improvement and gender role stress reduction, suggesting that this improvement may be achieved through alternative pathways such as behavioral activation, cognitive reappraisal of fatigue, or enhanced physical mastery. The progressive loading design and the short-duration, high-efficiency training format align well with university students’ actual circumstances and have good generalizability. However, the study sample was exclusively male university students (aged 18–22 years). While this intentional sample restriction is appropriate for addressing the research theme of male gender role stress, it limits the generalizability of the findings. Moreover, the use of the CD-RISC-10, a unidimensional resilience measure, represents a measurement limitation. Future research should explore the interactive relationship between psychological changes and gender role expectations in women undergoing similar high-intensity training, and consider using tools such as the MTQ48 (Fakhri et al., 2004) that better align with the four-dimensional model, or combine semi-structured interviews and other qualitative methods to capture the dimensional characteristics of resilience changes more comprehensively. In summary, maximal power cycling may serve as an effective and male-friendly intervention for improving resilience and alleviating gender role stress in male university students; however, its psychological mechanisms warrant further investigation in future research.

## Data Availability

The raw data supporting the conclusions of this article will be made available by the authors, without undue reservation.
