# Supplementary material for: Changes in resilience and masculine gender role stress following 8 weeks of maximal power cycling training in male university students: a single-group pre-post study
Source: Front Psychol. 2026 Jul 24;17:1876857. doi: 10.3389/fpsyg.2026.1876857 (PMC13447152; doi:10.3389/fpsyg.2026.1876857)
Supplement: Supplementary file 1 [file Supplementary_file_1.docx]

**Supplementary Material**

**2.3 Intervention Protocol**
The intervention protocol was described in accordance with the TIDieR (Template for Intervention Description and Replication) reporting guidelines to ensure reproducibility and transparency.

**2.3.1 Intervention Name**
8‑week progressive Wingate‑based sprint interval training (SIT)

**2.3.2 Rationale and Theoretical Framework**
This intervention was designed based on the classic Wingate sprint interval training protocol. The theoretical rationale included: (1) low‑volume high‑intensity interval training rapidly induces muscle metabolic adaptations, including enhanced mitochondrial biogenesis and increased glycogenolytic enzyme activity; (2) the “challenge–coping” cycle inherent in high‑intensity exercise may enhance psychological resilience through structured stress exposure; and (3) the time‑efficient nature of this exercise mode aligns with the limited time availability and low exercise participation common among university students. A progressive loading design was adopted (2 sessions/week during the acclimatisation phase, followed by 3 sessions/week during the intensification phase) to allow participants to gradually adapt to the training intensity, minimise initial discomfort, and achieve further improvements after adaptation.

**2.3.3 Intervention Materials and Equipment**
Primary equipment: Monark 824E mechanically braked cycle ergometer (Monark Exercise AB, Sweden). This ergometer uses a pendulum‑based mechanical resistance principle for loading and is equipped with a triple calibration function (pointer calibration, mechanical calibration, and electronic calibration).
Auxiliary equipment: Heart rate monitor (Polar H10, Finland) to record heart rate responses during training; stopwatch or timing software for precise control of sprint and recovery durations; and Rating of Fatigue (ROF) scale sheets to document subjective fatigue after each session.
Equipment calibration: Before each training session, the Monark 824E ergometer underwent mechanical zero calibration and resistance system calibration in accordance with the manufacturer’s specifications.

**2.3.4 Intervention Providers**
All training sessions were supervised by two postgraduate students majoring in exercise science who had received uniform training. The supervisors completed a 1‑week standardised training programme prior to the intervention, covering: (1) operation and calibration of the Monark 824E cycle ergometer; (2) standard operating procedures for the Wingate sprint test; (3) safety monitoring and emergency procedures; and (4) standardised verbal encouragement. At least one supervisor was present during each training session, responsible for equipment operation, timing, recording, and safety management.

**2.3.5 Intervention Procedures**
Training mode: A constant‑resistance mode was adopted. Each session consisted of four 30‑s “all‑out” sprint bouts, with 4 min of active recovery between sprints.
Resistance setting: The ergometer resistance was individualised based on each participant’s body weight, calculated as: Resistance (kg) = body weight (kg) × Cd (0.075). For example, a participant weighing 70 kg would have a resistance of 70 × 0.075 = 5.25 kg.
Cadence requirement: During sprints, participants were instructed to maintain the highest possible cadence (target ≥100 rpm). During recovery periods, cadence was maintained at 50–60 rpm.

**2.3.6 Intervention Procedure (per session)**
Each training session lasted approximately 30–35 min and was divided into three phases:

| Stage | Contents | Duration | Detailed description |
| --- | --- | --- | --- |
| Warm-up period | Low-intensity cycling without resistance | 5 minutes | Set the resistance to 0 kg, maintain a cadence of 60 rpm, switch on the machine and acclimatise to the training environment |
| Main training period | 4 × 30-second all-out sprints | Approximately 18–20 minutes | Each sprint: 0–30 seconds of all-out pedalling (maximum power output); immediately follow the sprint with a recovery period |
| Recovery period | Low-intensity active recovery | 4 minutes per session | Constant workload of 50 W, maintaining a cadence of 50–60 rpm, with a total of three recovery intervals (following the fourth sprint, proceed directly to the recovery phase) |
| Rest period | Low-intensity cycling | 5 minutes | Constant workload of 50 W, with cadence gradually reduced to 40 rpm to promote lactate clearance and heart rate recovery |

**2.3.7 Intervention Dose (When and How Much)**
Total intervention duration: 8 weeks (November 2025 – January 2026).
Training frequency:
– Weeks 1–3 (acclimatisation phase): 2 sessions per week (e.g., Tuesday and Thursday) → 6 sessions total.
– Weeks 4–8 (intensification phase): 3 sessions per week (e.g., Tuesday, Thursday, and Saturday) → 15 sessions total.
– Total: 21 sessions.

Dose per session:
– Number of sprints per session: 4
– Sprint duration: 30 s each
– Total sprint time: 120 s per session
– Total active recovery time: 12 min per session (3 intervals × 4 min)
– Total session time (including warm‑up and cool‑down): approximately 30–35 min

Total intervention dose summary:
– Total number of sprints: 84 (21 sessions × 4 sprints)
– Total sprint time: 42 min (84 sprints × 30 s)
– Total number of training sessions: 21

**2.3.8 Intervention Setting**
All training sessions were conducted in the Key Laboratory of Exercise Science and National Physical Fitness Promotion for the People in Ya’an City. Environmental conditions in the laboratory were maintained as follows:
– Temperature: 20–22 °C
– Relative humidity: 40%–60%
– Ventilation: air‑conditioning system and exhaust fans ensured adequate air circulation
– Lighting: standard indoor illumination
– The equipment was turned on and the laboratory environment was adjusted to the above standards 30 min before each session.

**2.3.9 Standard Operating Procedures**
To ensure consistency across sessions, the following standardised operating procedures were strictly followed:

Pre‑session:
– Participants were instructed to avoid vigorous exercise 24 h before each session.
– They were asked to refrain from eating a heavy meal 2 h before training.
– Up to 500 mL of water was allowed 1 h before training.
– Resting heart rate and blood pressure were measured and recorded before each session.
– Participants were questioned about any current symptoms (e.g., cold, muscle soreness, sleep deprivation). If any abnormality was reported, training was adjusted or postponed accordingly.

During‑session:
– A 3‑s countdown (“3‑2‑1‑Start”) was given before each sprint.
– Standardised verbal encouragement was provided by the supervisors during sprints (e.g., “Keep going!”, “Maintain the cadence!”, “Last 10 seconds!”), repeated every 5–10 s.
– Participants’ facial colour, sweating rate, and subjective responses were monitored in real time. Training was immediately terminated if any of the following occurred: participant’s request to stop; chest pain, severe dizziness, nausea, or dyspnoea; pallor or cyanosis; heart rate exceeding the age‑predicted maximum.
– Peak power (W) and mean power (W) were recorded immediately after each sprint.
– During the 3rd minute of each recovery period, the participant was asked to rate their fatigue using the ROF (0–10 scale).

Post‑session:
– Heart rate and blood pressure were measured and recorded after the cool‑down period.
– Total work output (kJ) and mean power (W) for the session were recorded.
– Participants were asked about any discomfort, and any adverse events were documented.

**2.3.10 Training Adherence and Data Recording**
Adherence criterion: Participants were required to complete at least 80% of the scheduled sessions (i.e., ≥17 out of 21 sessions) to be included in the final data analysis.

Adherence documentation: For each session, the supervisor recorded the following in a training log:
– Date and time of training
– Participant identification number
– Completion status (full completion / partial completion / absence)
– Reason for absence (if applicable)
– Peak power (W) and mean power (W) for each sprint
– Post‑session ROF score
– Any adverse events or unusual occurrences

Withdrawal criteria: Participants had the right to withdraw at any time, without obligation or penalty. Data from withdrawn participants were removed in accordance with the study protocol.

**2.3.11 Individualised Adjustments to the Intervention**
Individualisation was implemented in two main aspects:

(1) Resistance individualisation: The ergometer resistance was calculated based on each participant’s body weight (Resistance = body weight × 0.075), ensuring that individuals of different sizes experienced a relatively consistent relative load.

(2) Progressive loading: Training frequency gradually increased from 2 sessions/week in the acclimatisation phase to 3 sessions/week in the intensification phase, allowing participants to adapt progressively to the training intensity. If a participant reported an ROF ≥ 9 or exhibited marked discomfort during a session, the supervisor could, at their discretion, recommend a reduced‑dose session (e.g., omitting one sprint or extending recovery time), and such adjustments were documented in the training log.

**2.3.12 Safety Monitoring and Adverse Event Management**
Pre‑exercise screening: All participants completed the Physical Activity Readiness Questionnaire (PAR‑Q) and a health history questionnaire before enrolment. Individuals with any of the following were excluded: history of musculoskeletal injury, diagnosed psychiatric disorder, substance dependence, or history of cardiovascular disease.

Definition of adverse events:
– Minor adverse events: muscle soreness or fatigue occurring within 24 h after training, not requiring medical intervention.
– Moderate‑to‑severe adverse events: exercise‑related injuries, cardiovascular events, syncope, or other conditions requiring medical attention.

Adverse event management procedure:
– In the event of a moderate‑to‑severe adverse event during training, the session was immediately stopped and the laboratory emergency protocol was activated.
– Minor adverse events were recorded in the training log by the supervisor, and the participant’s suitability for continued participation was assessed before the next session.
– All adverse events were reported to the principal investigator within 24 h.

Adverse event report during the study: No moderate‑to‑severe adverse events occurred. Approximately 15% of participants reported leg muscle soreness 24–48 h after training, which resolved with adequate rest and stretching, and did not affect subsequent participation.

**2.3.13 Intervention Fidelity Checks**
To ensure that the intervention was delivered as planned, the following fidelity measures were implemented:
– Supervisors completed standardised training and passed an assessment prior to the intervention.
– A Standard Operating Procedure (SOP) checklist was used for every session to verify each step.
– The principal investigator conducted unannounced spot checks (1–2 sessions per month) to observe whether the supervisors’ procedures complied with the protocol.
– All training logs were reviewed weekly by the principal investigator to check data completeness and identify outliers.
